# Effect of reconstruction plate removal on dental implants in fibula flap mandibles: A biomechanical and clinical study

**DOI:** 10.1371/journal.pone.0343008

**Published:** 2026-02-23

**Authors:** Jianyao Huang, Junpeng Chen, Jinpeng Jiang, Huiming Wang, Dan Yu, Huiyong Zhu

**Affiliations:** 1 Department of Stomatology, the Fourth Affiliated Hospital of School of Medicine, and International School of Medicine, International Institutes of Medicine, Zhejiang University, Yiwu, China; 2 Department of Oral and Maxillofacial Surgery, The First Affiliated Hospital, Zhejiang University School of Medicine, Hangzhou, China; 3 Stomatology Hospital, School of Stomatology, Zhejiang University School of Medicine, Zhejiang Provincial Clinical Research Center for Oral Diseases, Key Laboratory of Oral Biomedical Research of Zhejiang Province, Cancer Center of Zhejiang University, Hangzhou, China; International Medical University, MALAYSIA

## Abstract

Vascularized autologous bone transplantation combined with implant restoration is a preferred method for functional mandibular reconstruction. However, there is currently no consensus on whether internal fixation devices, such as titanium plates and screws, must be removed during the reconstruction process. This study aimed to assess the biomechanical and clinical necessity of removing these fixation devices. Eight patients who underwent mandibular reconstruction with fibula flaps and subsequent dental implantation were included. The study utilized finite element analysis to simulate and compare biomechanical stress distributions in models where fixation devices were either retained or removed. The clinical outcomes including peri-implant health, masticatory efficiency, and oral health-related quality of life were evaluated through follow-up examinations and standardized questionnaires. The biomechanical analysis indicated that the maximum stress on the grafted fibula surrounding the implants was significantly lower in the retention group (42.07 ± 12.06 MPa) compared to the removal group (44.892 ± 14.80 MPa, *P* = 0.017*). Furthermore, a positive correlation was identified between the simulated stress levels on the implants and the severity of gingival bleeding (coefficient: 0.82, *P* = 0.013*). Clinically, while there were no significant differences in marginal bone loss between the two approaches, patients who retained the internal fixation devices reported better quality of life scores regarding functional limitations and physical pain. In conclusion, retaining internal fixation devices appears to reduce mechanical stress on the peri-implant fibula graft and is associated with improved patient-reported outcomes. These findings suggest that the routine removal of reconstruction plates may not be necessary and that retention can favour peri-implant health and patient comfort.

## Introduction

Trauma, tumors, and inflammation can lead to segmental defects of the mandible, significantly impacting patients’ quality of life and social functioning [1]. With advancements in microsurgery, vascularized autologous bone transplantation has emerged as the preferred method for reconstructing segmental defects in the oral and maxillofacial region [2]. The increasing popularity of functional jaw reconstruction [3], combined with improvements in surgical accuracy and the favorable bicortical structure of the fibula, has resulted in a growing interest among surgeons in implant and implant-overdenture restorations of the fibula [4].

**Fig 1:**
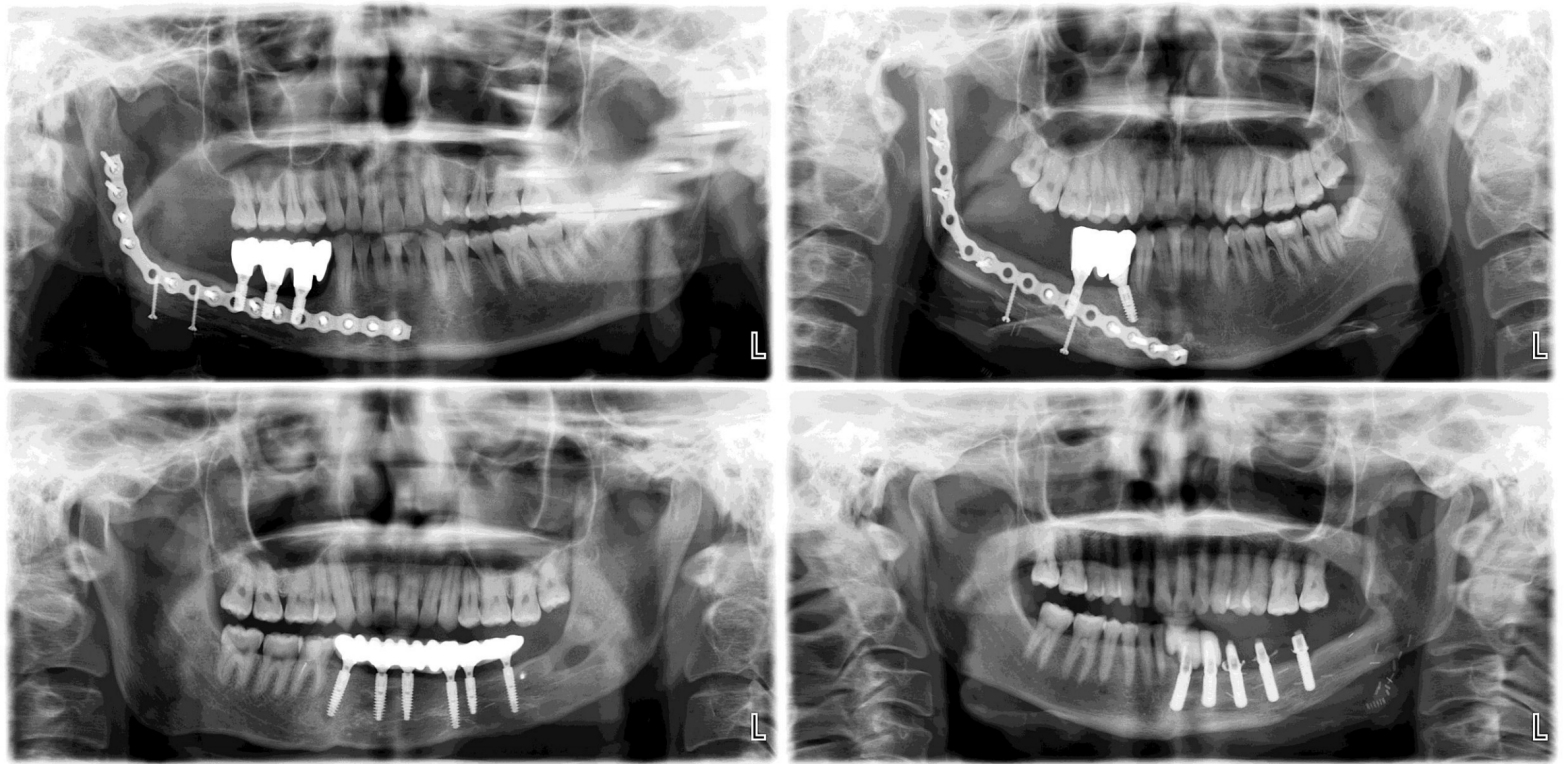
Implant restoration after mandibular reconstruction with fibula. Patients with internal fixation devices retained (upper) or removed (lower) are shown.

Previously, implant restoration following fibula reconstruction required the removal of internal fixation devices, including titanium plates and screws, to avoid interference with implant surgery [5]. However, the advent of digital surgical technology has allowed preemptive designing of implant-oriented fibula flaps. Implants may even be placed simultaneously during the reconstruction surgery based on the preoperative plan [6,7]. This development has eliminated the need to remove plates and screws solely because of positional interference. As shown in Fig 1, both plate-retention and plate-removal approaches to implant restoration have been performed at our hospital.

The necessity of plates and screws removal surgery is determined based on several factors. On one hand, retaining the internal fixation devices may lead to clinical complications, such as pain, infection, and titanium plate exposure [8], along with an increased risk of complex fractures resulting from stress concentration, particularly in trauma patients [9]. On the other hand, removal surgery is associated with an increased risk of nerve injury, skin scarring, and discomfort [10]. Additionally, patients undergoing mandibular defect reconstruction may experience psychological distress because of repeated surgeries under general anesthesia. Consequently, there is currently no consensus regarding the removal of plates and screws following jaw reconstruction [11]. This remains an important issue requiring further clarification to optimize functional mandibular reconstruction.

Finite element analysis (FEA) is the most widely used method for assessing biomechanical conditions during jaw reconstruction or implant restoration [12]. Regarding implants, clinical studies have reported significant bone absorption in peri-implant fibulas, particularly in the distal direction [13]. A FEA study reported significant effects of varying bone conditions on peri-implant bone stress distribution [14]. Although fibula has an advantageous bicortical structure [15] compared to the mandible, it has a smaller elastic modulus [16], which adds to the complexity of biomechanical studies in patients undergoing fibula reconstruction and implant restoration. Currently, there is a lack of theoretical or clinical evidence regarding whether removal of internal fixation devices leads to more favorable biomechanical stress distribution and improved functional restoration.

Based on these considerations, this study used FEA to analyze stress distribution in patients undergoing implant restoration after fibula reconstruction. Two biomechanical models, with the internal fixator either retained or removed, were compared. Peri-implant health and functional recovery in both groups were then comprehensively and systematically evaluated through clinical follow-up. We hypothesized that retention of the internal fixator would result in more favorable stress distribution in the grafted fibula and, consequently, improved peri-implant health.

## Methods

Patients who underwent mandibular reconstruction followed by implant restoration at the First Affiliated Hospital of Zhejiang University School of Medicine between January 1, 2017, and December 31, 2022, were included in this study. Raw data for research purposes were accessed on February 1, 2024. The inclusion criteria were as follows: (1) at least three remaining teeth on the healthy side of the mandible; (2) a stable occlusal relationship; (3) adequate postoperative recovery without disease recurrence; and (4) completion of implant restoration with regular use for more than one year. Written informed consent was obtained from all participants, all patient data were anonymized to ensure confidentiality, and the study posed minimal risk to participants. The study was approved by the Medical Ethics Committee of the First Affiliated Hospital of Zhejiang University School of Medicine (reference number: 2019-1556-2).

Cone Beam Compute Tomography (CBCT) images were acquired for all participants using the NewTom VG system (NewTom, Bologna, Italy), covering the region from the hyoid bone to the articular fossa with a field of view of 16 × 16 cm and a slice thickness of 0.3 cm. Digital Imaging and Communications in Medicine (DICOM) files were imported into Mimics Research 21.0 (Materialise, Leuven, Belgium) to generate three-dimensional reconstructions of the entire mandible, including bone, teeth, and implant-supported crowns. The implants, titanium plates, and screws were excluded from the reconstruction models. The reconstructed mandible was then exported in stereolithography format and imported into Geomagic Studio 2013 software (3D Systems Corp., Rock Hill, SC, USA). Following modification, denoising, and surface partitioning, the model was exported in standard for the exchange of product model data (STP) format. STP files were then imported into the SOLIDWORKS 2020 software (Dassault Systems, Paris, France) to design the implants and abutments based on the actual anatomy.

The mandible was segmented into three parts: fibula segment, healthy-side segment, and affected-side segment, based on the actual osteotomy and reconstruction methods. This model was named the “removal model” and saved in Parasolid Text File (X-T) format. The titanium plate was designed in SOLIDWORKS software with a thickness of 2 mm, a width of 6 mm, and a hole diameter of 3 mm. Titanium screws, with a head diameter of 3.5 mm and a shaft diameter of 2.4 mm, were generated based on three-dimensional scanning of the actual components. Finite element analysis was conducted using a virtual model of the patient-specific mandible and fibula, in which titanium plates and screws were positioned to replicate clinical internal fixation under real surgical conditions. This model was named the “retention model” and saved in X-T format.

**Fig 2:**
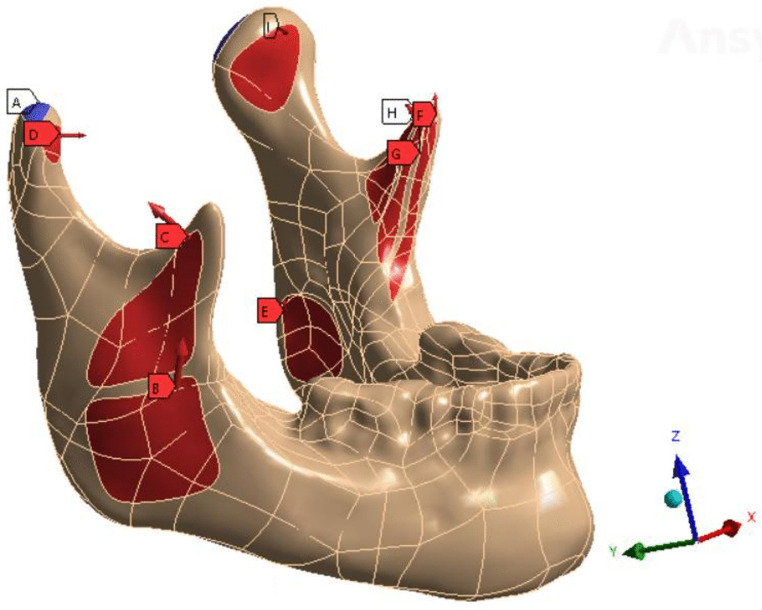
Masticatory attachment areas of mandible. A: constrained; B: superficial masseter; C: deep masseter; D: inferior lateral pterygoid; E: medial pterygoid; F: anterior temporalis; G: middle temporalis; H: posterior temporalis; I: contralateral inferior lateral pterygoid. The red arrows represent the direction of muscle force.

The X-T format model was imported into Ansys Workbench 2022R1 software (Ansys, Inc., Canonsburg, PA, USA). Static structure analysis was selected and individual structures, including bones, natural teeth, implants, denture crowns, and titanium plates, were individually meshed. A tetrahedral mesh structure with a size of 0.1 mm was preferentially used. Entities containing fine structures, such as titanium screws or bone segments fixed using screws, could be meshed with smaller sizes according to the actual requirements. The biomechanical parameters of mandibular bone were calculated based on the cortical bone material. The material properties of each structure are presented in Table 1 [17–20]. All materials were assumed to be homogeneous, isotropic, and linearly elastic. To simulate the effect of muscle forces, up to 14 masticatory muscle attachment areas were delineated on the model according to previous anatomical studies (Fig 2) [21]. The prosthetic side was designated as the working side to simulate the masticatory state of the implant denture, with the denture molars and bilateral condyles constrained to fixed positions (Table 2) [21]. The models represented patients with good bone healing more than 2 years after the surgery. Healing and reattachment of the masticatory muscles were assumed to have reached an ideal state. A 10-node quadratic tetrahedral element (C3D10) was used to mesh the geometric model. Mesh sensitivity analysis was performed by comparing three finite element models with mesh sizes of 0.2 mm, 0.5 mm, and 1 mm. The relative difference in implant von Mises stress across all mesh sizes was less than 5%; therefore, a mesh size of 1 mm was selected for bone structures, and 0.2 mm was selected for titanium components and implant screws. Interfacial interactions between the screw surfaces and adjacent structures were defined using a tie constraint. The equivalent von Mises stress distribution, total displacement, and equivalent strain for each structure were analyzed using Workbench, and the maximum value and distribution of the results were calculated.

**Table 1:** Material properties of each structure in the FEA mandible model.

Structure	Elasticity modulus (MPa)	Poisson’s ratio
Titanium plate and screws	110,000	0.33
Fibula	7,300	0.3
Mandible	13,700	0.3
Natural tooth	20,000	0.3
Crown of implants	205,000	0.3
Implant and abutment	105,000	0.33

**Table 2:** Masticatory muscle forces on the FEA mandible model.

Clenching tasks	Muscle side	Direction	Muscular force (N)							Boundary condition
			SM	DM	MP	AT	MT	PT	ILP	
**Right molar**	Right	Force	137.1	58.8	146.9	115.4	63.1	44.6	20.1	Right molars constrained
		Fx	−28.4	−32.1	71.4	−17.2	−14.0	−9.3	12.6	
		Fy	121.2	44.5	116.1	114	52.8	21.1	−3.5	
		Fz	57.4	−21.0	54.8	5.1	−31.5	−38.1	15.2	
	Left	Force	114.2	49	104.9	91.7	64	29.5	43.5	
		Fx	23.6	26.7	−51.0	13.7	14.2	6.1	−27.4	
		Fy	101	37.1	83	90.5	53.6	14	−7.6	
		Fz	47.9	−17.5	39.1	4	−32.0	−25.2	32.9	
**Left molar**	Right	Force	114.2	49	104.9	91.7	64	29.5	43.5	Left molars constrained
		Fx	−23.6	−26.7	51	−13.7	−14.2	−6.1	27.4	
		Fy	101	37.1	83	90.5	53.6	14	−7.6	
		Fz	47.9	−17.5	39.1	4	−32.0	−25.2	32.9	
	Left	Force	137.1	58.8	146.9	115.4	63.1	44.6	20.1	
		Fx	28.4	32.1	−71.4	17.2	14	9.3	−12.6	
		Fy	121.2	44.5	116.1	114	52.8	21.1	−3.5	
		Fz	57.4	−21.0	54.8	5.1	−31.5	−38.1	15.2	

**Fig 3:**
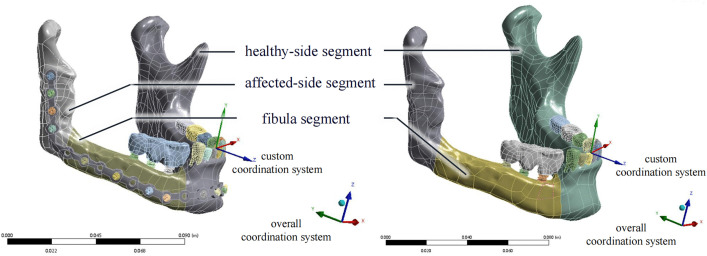
Internal fixation retention and removal models of a single patient. The three model components (the fibula segment, healthy-side mandible, and affected-side mandible) are shown.

DM: deep masseter; SM: superficial Masseter; ILP: inferior lateral pterygoid; MP: medial pterygoid; AT: anterior temporalis; MT: middle temporalis; PT: posterior temporalis. The lower incisor point was defined as the coordinate origin, and a line bisecting the mandibular dentition and passing through the origin on the lower occlusal plane was defined as the x-axis, with the forward direction representing positive F_x_ values. A line perpendicular to the x-axis, passing through the origin on the lower occlusal plane, was defined as the z-axis, with the left direction representing positive F_z_ values. A line perpendicular to the lower occlusal plane, passing through the origin, was defined as the y-axis, with an upward direction representing positive F_y_ values. The customized coordinate system is shown in Fig 3.

In addition to FEA, the subjects also underwent clinical follow-up assessments. CBCT examinations were conducted immediately (T0), 6 months (T1), and 12 months (T2) after implantation and at the final follow-up (T3). Defect depth (DD) was defined as the distance between the marginal bone-implant junction and the implant-abutment junction, measured on CBCT. Marginal alveolar bone height changes at different time points were recorded and compared with T0, and the differences were defined as marginal bone loss (MBL). Measurements were performed using NNT Viewer 6.0 (New Tom, Bologna, Italy), ensuring that the full length of the implant and central screw hole were visible in the image, and the complete shape of the implant could be clearly distinguished without any overlapping [22]. DD and MBL were measured at both medial and distal sites for each subject.

During the final follow-up, peri-implant health status was assessed by the a single attending physician using the following indicators and recording criteria: (1) modified plaque index (mPLI), with a score of 0 indicating no plaque, 1 indicating plaque detectable on probing of the denture surface, 2 indicating plaque visible on visual examination, and 3 indicating significant plaque and materia alba accumulation; (2) modified sulcus bleeding index (mSBI), where a score of 0 indicated no bleeding on probing around the implant, 1 indicated punctate bleeding, 2 indicated linear bleeding, and 3 indicated bleeding extending beyond the gingival sulcus; and (3) peri-implant probing pocket depth (PPD), with a PPD of 5 mm serving as a threshold between peri-implant tissue health and inflammation. During implant examination, a total of six buccal-lingual sites were assessed, and the maximum PPD and mSBI values were recorded. Since implants were combined with union crowns for each patient, only the maximum mPLI of the union crown was recorded.

Masticatory efficiency was evaluated using ViewGum software (dHAL Software, Kifissia, Greece), an image analysis software [23]. Specimens were prepared from Wrigley gum (Wm. Wrigley Jr., Co., Chicago, IL, USA) in two colors, with strips cut from both colors and manually joined together to create a test strip measuring 30 mm × 20 mm × 4 mm. The participants were instructed to chew the gum on the implant denture for 1 minute. The chewed gum was then placed into an oral instrument box, flattened into a rectangular shape according to the grid of the box, and photographed using a camera under consistent parameters and lighting conditions. ViewGum software was used to analyze the images according to established protocols [24]. The variance of the hue component was considered the measure of mixing, and standard deviation of hue (SDHue) was calculated and compared between groups [25].

The patients were further interviewed at T0 and T3 using the Oral Health Impact Profile-Implant (OHIP-I) [26] to assess oral health-related quality of life (Table 3). The questionnaire comprised 14 items across three areas: functional limitation, physical pain and discomfort, and psychological and social impact. Each item was self-evaluated on a five-point scale (0 = never, 1 = rarely, 2 = sometimes, 3 = often, and 4 = always). The sum of the item scores was calculated to obtain total scores for each area and the entire questionnaire, with higher scores indicating a greater impact on the individual in the respective area.

**Table 3:** OHIP-I for partially edentate patients with implant-supported prostheses.

Areas	Items
Functional limitation	
	Q1 Difficulty chewing
	Q2 Trouble pronouncing words
	Q3 Appearance affected
	Q4 Food catching
	Q5 Dentures not fitting
Physical pain and discomfort	
	Q6 Painful aching
	Q7 Discomfort when eating
	Q8 Discomfort from dentures
	Q9 Avoidance of eating
Psychological and social impact	
	Q10 Feeling worried
	Q11 Self-conscious
	Q12 Difficult to relax
	Q13 Concentration affected
	Q14 Life unsatisfying due to oral condition

SPSS 27.0 (IBM Corp., Armonk, NY, USA) was used for data analysis, with all data assessed for normal distribution and homogeneity of variance. Due to the small sample size, the Wilcoxon signed-rank test was used to compare von Mises stress between the fixation retention and removal finite element models for the same patient. Probing pocket depth and questionnaire scores were analyzed using the Mann–Whitney U test, whereas probing pocket depth and modified sulcus bleeding index were compared using a group t test. Spearman correlation analysis was performed to assess associations between clinical peri-implant health indicators (modified plaque index, modified sulcus bleeding index, and probing pocket depth) and implant von Mises stress under patient-specific conditions. The significance level was set at α = 0.05 (two-tailed) for finite element analysis results. For all clinically relevant outcomes, Holm–Bonferroni–corrected *P* values were used to determine statistical significance. *P* values marked with an asterisk were considered statistically significant.

## Results

The study included a total of 8 patients, comprising 6 males and 2 females, with an average age of 30.9 ± 12.83 years. All patients recovered successfully postoperatively without recurrence. All fibula flaps survived without complications, including infection, loosening, or exposure of titanium plates and screws, and exhibited satisfactory bone healing. Based on personal preferences, three of the patients underwent removal of the internal fixator, while five did not undergo removal (Table 4). All participants in this study had benign mandibular tumors, including ossifying fibroma in Patient No. 5 and ameloblastomas in the remaining patients. None of the patients received postoperative radiotherapy, chemotherapy, or other adjuvant therapies. Patient 2 had an implant removed 4 years after implantation due to peri-implant inflammation, with the remaining two implants surviving until the final follow-up. The overall survival rate of implants across all patients was 96.67%.

**Table 4:** Basic information of patients at follow-up.

NO.	Group	Sex	Age	Resection range	Number of implants	Follow up time
1	Retention	Male	25	44-right coracoid	3	4.5y
2	Retention	Male	24	44-right sigmoid notch	3	5y
3	Retention	Male	19	44-right condyle	2	4y
4	Retention	Male	26	34-left sigmoid notch	2	6y
5	Retention	Female	56	44-right angle of mandible	3	3y
6	Removal	Male	34	42- left angle of mandible	5	2.5y
7	Removal	Fmale	43	41-left angle of mandible	5	2.5y
8	Removal	Male	20	45-left angle of mandible	6	6y

FEA models of both internal fixation removal and retention were established for each patient, resulting in a total of 16 FEA models. An example of the model is depicted in Fig 3.

**Fig 4:**
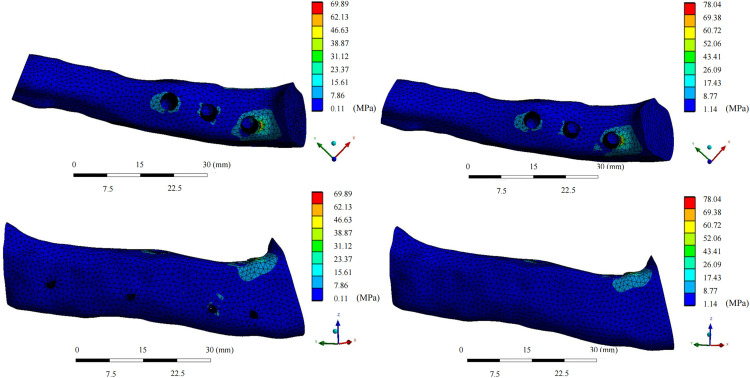
Equivalent von Mises stress distribution for the grafted fibula after implant restoration. Upper view of the fibula in the retention group; upper view of the fibula in the removal group; front view of the fibula in the retention group; and front view of the fibula in the removal group.

**Fig 5:**
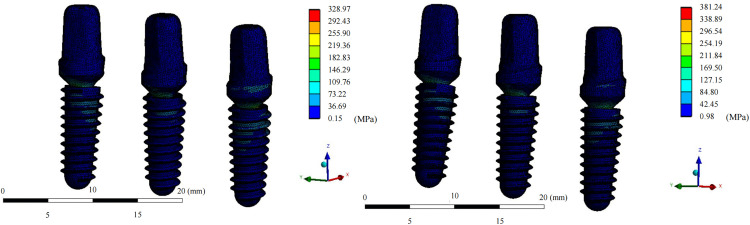
Equivalent von Mises stress distribution for the implant and abutment. Diagrams of the implant and abutment models in the retention and removal groups.

**Fig 6:**
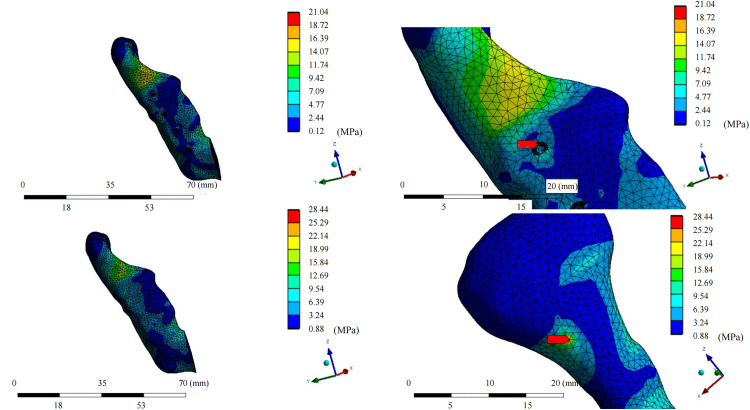
Equivalent von Mises stress distribution diagram of the affected-side mandible. Mandible in retention group; mandible in retention group with the maximum stress (red arrow) located at the interface between the titanium screw and bone; mandible in removal group; mandible in removal group with the maximum stress (red arrow) located at posterior aspect of the ascending branch of the mandible.

**Fig 7:**
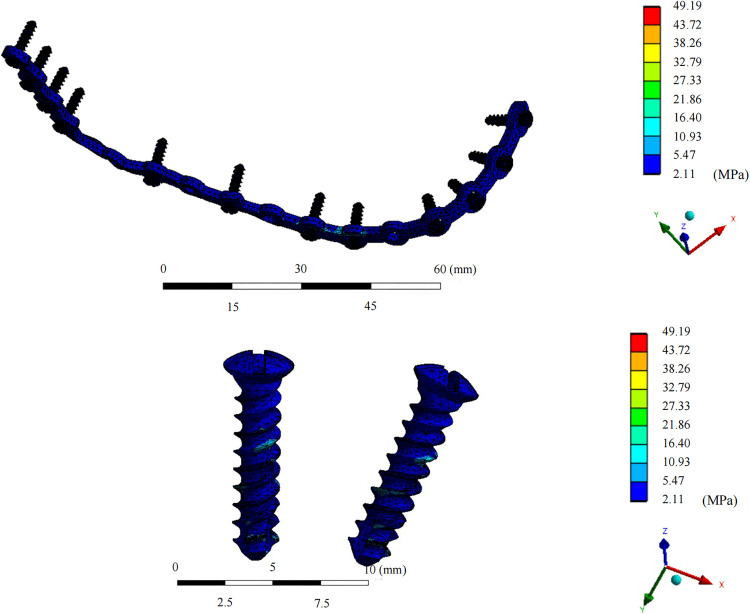
Equivalent von Mises stress distribution diagram of titanium plate and screws. The overall plate–screw assembly and magnified views of two representative screws are shown.

In the fixation device retention group, the maximum von Mises stress for the grafted fibula with the implant was 42.07 ± 12.06 MPa, which was lower than that in the fixation device removal group (44.892 ± 14.80 MPa; median difference, 2.40 [95% CI, 0.19 to 6.28), Z = −2.38, *P* = 0.017*). The maximum stress for each patient was present at the osseous interface between the implant and the fibula (Figs 4 and 5). On the healthy-side mandible, the mean maximum stress in the retention group was 48.92 ± 35.04 MPa, compared to 31.56 ± 14.58 MPa in the removal group (median difference, −10.08 [95% CI, −47.20 to −0.36], Z = −1.96, *P* = 0.0499*). The maximum stress on the healthy-side mandible occurred at the interface between the jaw and the retained titanium screws in three models. On the affected side, the mean maximum stress in the retention group was 33.71 ± 18.92 MPa, while that in the removal group was 31.52 ± 21.10 MPa, with no statistically significant difference. In both groups, the maximum stress on the affected side appeared at the interface between the jaw and the retained titanium screws in five models (Figs 6 and 7). The mean maximum strain of the healthy-side mandible was higher in the retention group (0.0053 ± 0.0050 m/m) than in the removal group (0.0027 ± 0.0013 m/m), indicating a tendency toward increased strain with fixation retention. However, there were no significant differences in the remaining biomechanical indices between the two groups. The complete von Mises stress, strain, and deformation data for each entity of the models in both groups are presented in Tables 5 and 6.

**Table 5:** Von Mises stress, total displacement, and strain of the fibula segment and implant.

		Grafted fibula	*P* value	Implant	*P* value
Maximum von Mises stress (MPa)	Retention group	42.07 ± 12.06	0.017*	192.58 ± 95.22	0.093
	Removal group	44.92 ± 14.80		203.73 ± 102.44	
Maximum displacement (mm)	Retention group	0.066 ± 0.016		0.027 ± 0.014	
	Removal group	0.068 ± 0.020		0.028 ± 0.017	
Maximum strain (m/m)	Retention group	0.0058 ± 0.0017		0.0020 ± 0.00089	
	Removal group	0.0063 ± 0.0021		0.0021 ± 0.00091	

**Table 6:** Von Mises stress, total displacement, and strain of the healthy- and affected-side mandible.

		Healthy-side mandible	*P* value	Affected-side mandible	*P* value
Maximum von Mises stress (MPa)	Retention group	48.92 ± 35.04	0.0499*	33.71 ± 18.92	0.89
	Removal group	31.56 ± 14.58		31.52 ± 21.10	
Maximum displacement (mm)	Retention group	0.15 ± 0.089		0.080 ± 0.040	
	Removal group	0.16 ± 0.095		0.077 ± 0.036	
Maximum strain (m/m)	Retention group	0.0053 ± 0.050		0.0032 ± 0.0022	
	Removal group	0.0027 ± 0.0013		0.0029 ± 0.0018	

The MBL and DD values for the patients are presented in Table 7, with patient serial numbers corresponding to those in Table 4. Patients 1–5 had internal fixation retained, while patients 6–8 had the internal fixation removed. The mean DD for all patients was 1.50 ± 0.64 mm, indicating good peri-implant health, with no significant difference between the two groups.

**Table 7:** Marginal bone loss (MBL) and defect depth (DD) of patients at follow-up.

	T1		T2		T3	
**No.**	Mesial MLB	Distal MLB	Mesial MLB	Distal MLB	Mesial DD	Distal DD
1	0.2	0.5	0.3	0.8	0.4	1
2	0	0	0.5	2.5	0.5	2.5
3	0	0	0	0	0	0.7
4	0.6	0.5	0.7	0.8	1.3	1.5
5	0	0.6	0.2	0.8	0.3	2
6	0.2	0.3	0.2	0.5	0.6	0.8
7	0	0.3	0.4	1.3	0.9	2
8	0	0	0	0.6	0	1.5
Mean ± SD	0.13 ± 0.21	0.28 ± 0.25	0.29 ± 0.24	0.91 ± 0.74	0.49 ± 0.44	1.50 ± 0.64

In the retention group, the mean mPLI, mSBI, and PPD values were 1.00 ± 0.71, 1.58 ± 0.79, and 3.67 ± 0.78, respectively. The corresponding values in the removal group were 1.33 ± 1.53, 1.37 ± 0.40, and 3.00 ± 0.97, respectively. The maximum PPD in both groups was 5 mm, which did not exceed the threshold for health status. Correlation analysis demonstrated a positive association between maximum implant equivalent stress and the modified sulcus bleeding index, with a correlation coefficient of 0.82 (95% CI, 0.25 to 0.97; *P* = 0.013*). Although no significant associations were observed for the other indicators, this finding suggests that higher simulated implant stress may be associated with increased probing bleeding severity.

Regarding masticatory efficiency, the SDHue was 0.050 ± 0.031 in the retention group and 0.031 ± 0.0087 in the removal group. Although the SDHue score of the removal group was lower, suggesting higher masticatory efficiency, the difference was not statistically significant. Questionnaire responses showed that the functional limitation score at T0 was lower in the retention group (4.00 ± 1.00) than in the removal group (6.67 ± 1.53; median difference, −3.00 [95% CI, −5.00 to 0.00]; Z = −2.00, *P* = 0.046). This pattern remained consistent at T3. At T3, the physical pain and discomfort score was also lower in the retention group (0.40 ± 0.89) compared with the removal group (2.67 ± 1.53; median difference, −3.00 [95% CI, −4.00 to −1.00]; Z = −2.07, *P* = 0.039) (Table 8).

**Table 8:** OHIP-I questionnaire scores of patients at follow-up.

Restoration completed (T0)				
Group	Functional limitation	Physical pain and discomfort	Psychological and social impact	Total
Retention group	4.00 ± 1.00	3.00 ± 3.46	2.00 ± 3.08	9.00 ± 6.96
Removal group	6.67 ± 1.53	4.00 ± 1.00	4.33 ± 3.79	15.00 ± 6.08
Last follow up (T3)				
Retention group	3.00 ± 1.00	0.40 ± 0.89	1.00 ± 1.22	4.40 ± 2.70
Removal group	4.67 ± 2.52	2.67 ± 1.53	4.67 ± 3.21	12.00 ± 7.00

## Discussion

Reducing bone resorption is crucial for ensuring the long-term success of implant restoration following mandibular reconstruction [27]. Excessive mechanical loads [28] and peri-implant inflammation [29,30] are the main causes of peri-implant bone resorption. Previous studies have used equivalent stress values as thresholds for bone injury, with reported damage thresholds ranging from 50 to 190 MPa for different bone types [31–33]. Moreover, stress values exceeding 616 MPa may lead to the fracture of the titanium reconstruction plate [34]. FEA serves as an effective method for studying jaw biomechanics [35]. Given the significant anatomical variability among patients who have undergone jaw reconstruction, 16 specific FEA models were established. The maximum von Mises stress for the bone was 20–50 MPa, while the stress values for implants ranged from 100 to 300 MPa, with the values being consistent with previous studies [36–38]. The analysis results demonstrated that the equivalent stress on the grafted fibula surrounding the implant was lower in the retention group compared to the removal group (*P* = 0.017*). However, areas of stress concentration were observed around the retained titanium screws in the retention group, particularly in the healthy-side mandible, where the difference was statistically significant (*P* = 0.0499*). The equivalent stress around the implant, retained titanium screws, and the titanium plate itself did not exceed the damage threshold. Consequently, subsequent clinical reviews did not reveal significant differences in MBL, and the titanium plate and screw fixation remained intact. Frost et al. [39,40] suggested that bone strain could serve as a secondary index for predicting intraosseous implant loosening. When the bone strain around the implant surpasses 3000–4000 micro-units, minor bone damage requiring remodeling or repair may occur. The maximum bone strain for some models in our study exceeded this threshold, suggesting that efforts should be made clinically to minimize stress concentration, thereby reducing bone strain and mitigating the risk of implant loosening [41].

Regarding peri-implantitis, another significant cause of bone resorption, periodontal health indicators showed no significant differences between the two groups. However, patients with higher implant stress levels exhibited significantly greater mSBI values. Previous studies have demonstrated that mechanical stress can trigger cytological changes in periodontal tissues, influencing the absorption and remodeling of periodontal fibers and alveolar bone [42,43]. Previous studies have demonstrated the existence of a remodeling threshold for alveolar bone. Both stress shielding [44] and excessive mechanical stress exceeding 50 MPa[33] can disrupt normal bone remodeling, ultimately leading to peri-implantitis. Based on the findings of our finite element analysis, excessive bone stress represents the primary concern in the present study. High compressive stress has been shown to diminish the viability of periodontal fibroblasts and osteoblasts [45], potentially leading to pathological inflammation [46]. The mechanisms of mechanically induced bone resorption include activation of the ERK pathway [47], the RANKL/OPG pathway [48], and the NFκB pathway [49]. However, factors such as individual differences in oral hygiene, smoking history, and postoperative maintenance and follow-up practices can independently influence the development of peri-implant disease, thereby confounding the direct relationship between implant stress and the mSBI. While the mean stress for implants in the retention group was slightly lower, the difference was not statistically significant. Furthermore, MBL, DD, and the simulated stress of peri-implant fibula also showed no significant correlations. This may be attributed to the fact that bone resorption around the implant is influenced by various other factors, such as the occlusal relationship [50] and chewing habits [51], making MBL and DD evaluations less reliable. Additionally, FEA analysis revealed a large inter-group deviation in implant stress, whereas the inter-group deviation in fibula stress was relatively small, which might explain the lack of a clear correlation between fibula stress and clinical manifestations.

The patient’s quality of life is an important consideration for determining the necessity of internal fixation removal. According to the questionnaire responses, patients in the removal group reported poorer outcomes in terms of functional limitation at T0, along with physical pain and discomfort at T3. Previous studies have shown that plate removal surgery may be associated with postoperative complications [52]. In addition, removal procedures performed under general anesthesia may increase patients’ psychological burden, potentially contributing to psychological disorders [53]. Consistent with these findings, our results suggest that plate removal surgery may have negative impacts; however, these observations require confirmation in larger-scale studies.

All patients included in this study underwent reconstruction using standard reconstruction plates, and micro titanium plates were not used. However, previous studies have reported that patients who received miniplates for fibula fixation experienced fewer complications after plate removal surgery [54]. In addition, miniplates have been associated with smaller healing gaps and more balanced stress distribution compared with reconstruction plates [13]. Therefore, miniplates may represent a more suitable option for internal fixation in patients undergoing dental implantation.

This study has several limitations. First, the sample size was small, rendering the statistical results susceptible to the influence of extreme values. A post hoc power analysis yielded a statistical power of 0.59, indicating limited robustness. Therefore, the findings cannot be directly generalized to a broader population or used to draw definitive statistical conclusions and should be interpreted strictly as exploratory and hypothesis-generating. Future studies will aim to expand the sample size by enrolling a larger number of eligible patients. Second, although finite element analysis was used to simulate biomechanical behavior, certain factors influencing biomechanics, such as patients’ dietary habits, could not be incorporated into the models. While patient-specific bone and tooth geometries were derived from imaging data, muscle force parameters were obtained from the literature [21], which may have introduced discrepancies. In addition, uniform material properties were assigned to the jawbone in the models [55,56]; compared with models incorporating heterogeneous bone quality [35,57], this approach may be less accurate. Furthermore, complete osseointegration between the implant and the grafted bone was assumed, whereas incomplete osseointegration in clinical practice could result in higher interfacial stress and reduced stability compared with simulated conditions.

Finally, as this was a retrospective study without random group allocation, interpatient variability may have affected the generalizability of the results. Future research should include prospective studies with larger cohorts and longer follow-up periods, with patient grouping based on clinical characteristics and surgical factors to minimize confounding. More refined biomechanical models that better simulate physiological oral movements will be developed, and additional assessment tools, such as occlusal force gauges and T-scan analysis, along with multimodal imaging evaluations, will be applied to comprehensively assess oral function. Moreover, standardized decision criteria or algorithms for determining whether to remove internal fixation devices should be established to optimize clinical outcomes. Finally, biodegradable plates are increasingly being used in fibula reconstruction [58], and their integration with biomechanical modeling for preoperative planning represents a promising direction for future research [59].

The FEA method proved useful in treatment planning for determining whether internal fixation devices should be removed. Compared with the removal group, the retention group exhibited lower equivalent stress in the grafted fibula surrounding the implants. However, areas of stress concentration were observed around retained titanium screws, with the maximum stress in the healthy-side mandible being significantly higher than that in the removal group. A significant positive correlation was identified between implant stress and the mSBI, and patients in the retention group exhibited slightly lower implant stress overall. Patients who underwent fixation removal reported subjective symptoms, including functional limitation, physical distress, pain, and discomfort, although these differences did not remain statistically significant after correction for multiple comparisons. Overall, retention of internal fixation devices may help reduce mechanical stress on the peri-implant grafted fibula and is associated with a trend toward fewer surgical complications.

## Clinical implications and recommendations

This study clarifies the clinical trade-offs involved in internal fixation management following fibular graft–based mandibular reconstruction. Retention of the internal fixator may reduce peri-implant fibular stress and the risk of additional surgical complications, whereas fixator removal may be appropriate for selected patients with poor native mandibular bone quality or in whom mechanical interference is a concern.

Based on our findings, retention of the internal fixator appears to be the preferred strategy for patients without subjective discomfort. In contrast, fixator removal may be considered in cases of recurrent inflammation around the titanium plate, screw loosening, progressive bone resorption, or pre-existing abnormal alveolar bone density. Preoperative digital planning is recommended to ensure that titanium plates and screws do not interfere with subsequent implant placement. In addition, regular postoperative follow-up, including radiographic evaluation, should be performed to monitor for screw loosening or graft resorption. Patients should also be encouraged to engage in appropriate functional exercises to facilitate postoperative recovery.

## Supporting information

S1 File Supporting information. (XLSX)

## Abbreviations

FEA: Finite element analysis
OHIP-I: Oral Health Impact Profile-Implant
PPD: Probing pocket depth
DD: Defect depth
MBL: marginal bone loss
mPLI: modified plaque index
mSBI: modified sulcus bleeding index
STP: Standard for the exchange of product model data
CBCT: Cone-beam computed tomography
DICOM: Digital imaging and communications in medicine
X-T: Parasolid Text File
SDHue: Standard Deviation of Hue
